# Effect of aesthetic crown lengthening on life quality and self‐esteem: 2‐year study

**DOI:** 10.1002/cap.70030

**Published:** 2026-05-22

**Authors:** Leticia S. Arroteia, Camila S. Stolf, Hélvis E. S. Paz, Thais F. M. Paschoal, Mabelle F. Monteiro, Márcio Z. Casati, Mauro P. Santamaria, Renato C. V. Casarin

**Affiliations:** ^1^ Department of Prosthodontics and Periodontics Periodontics Division Piracicaba Dental School, University of Campinas Piracicaba Brazil; ^2^ University of Kentucky College of Dentistry Lexington Kentucky USA; ^3^ Department of Periodontics and Endodontics Periodontic Division School of Dental Medicine University at Buffalo Buffalo New York USA

**Keywords:** crown lengthening, esthetics, gingiva, plastic surgery, quality of life, self‐esteem

## Abstract

**Background:**

A gummy smile can result from several factors, often leading to a disharmonious appearance and causing physical and psychological discomfort for the patient. This study aimed to evaluate changes in self‐esteem and quality of life for patients, as well as the recidivism rate, following aesthetic crown lengthening (ACL) surgery.

**Methods:**

Twenty‐two periodontally healthy patients who expressed interest in undergoing ACL and met the procedural indications were selected. Before surgery, at 30 days, and 2 years postoperatively, the Oral Health Impact Profile – 14 items (OHIP‐14) and the Rosenberg Self‐Esteem Scale were used to assess patients' perceptions of quality of life and self‐esteem. Crow dimensions, gingival phenotype, and the recidivism rate were assessed.

**Results:**

ACL significantly improved the psychosocial parameters of patients (*p* < 0.05). Both self‐esteem and quality of life showed improvement 30 days post‐surgery (*p* = 0.02 and 0.00024, respectively). After 2 years, further improvements were observed in the self‐esteem index compared to the 30‐day results (from 11 [interquartile range {IQR}: 8.0–14.0] to 14 [IQR:12–15, *p* < 0.05]) and in the OHIP score (from 19 [IQR:14–27] to 6 [IQR: 0.0–8.0]). The clinical crown length established 30 days after surgery remained stable after 2 years (0.82 ± 0.11).

**Conclusions:**

ACL can enhance patients' quality of life and self‐esteem, serving as a valuable procedure in aesthetic periodontics.

**Plain language summary:**

A “gummy smile,” where too much gum tissue shows when a person smiles, can affect both appearance and confidence. This study looked at whether aesthetic crown lengthening surgery (a procedure that reshapes the gums and exposes more of the teeth) could improve how people feel about themselves and their daily lives. We followed 22 patients who underwent this surgery and measured their self‐esteem and quality of life before the procedure, 1 month later, and again 2 years after the procedure. Results showed that patients felt noticeably better about their smiles and overall quality of life just 1 month after surgery. Even more encouraging, these benefits continued to grow over the course of 2 years, with patients reporting higher self‐esteem and fewer problems related to their oral health. Importantly, the changes achieved by the surgery—such as the new tooth and gum proportions—remained stable, with no return of the gummy smile. These findings suggest that crown lengthening is not only an effective and lasting solution for improving the appearance of a gummy smile but also has meaningful, long‐term benefits for patients’ confidence and well‐being.

## INTRODUCTION

Crown lengthening surgery is commonly indicated in various clinical scenarios, including facilitating restorative procedures, managing subgingival caries, and addressing aesthetic concerns.^1^ It encompasses both functional and aesthetic components and may, in some cases, require a multidisciplinary approach to achieve optimal outcomes.^2^ When performed for aesthetic reasons, particularly in the treatment of a gummy smile, the procedure is referred to as aesthetic crown lengthening (ACL). The goal of ACL is to reposition the supracrestal attachment tissues in a more apical direction, with the primary objective of creating a more harmonious and balanced smile.^3^

A gummy smile is a result of several factors, including excess gingival tissue covering the teeth, excessive vertical maxillary dimension, and/or hyperactivity of the upper lip.^4^ Excessive gingiva is primarily caused by altered passive eruption, where the physiological apical migration of the gingival margin to a level near the cementoenamel junction (CEJ) does not occur after tooth eruption.^5^ This condition leads to a clinically shorter crown than the anatomical crown, resulting in teeth that appear short, square, and wide.^6^ Such an appearance can create a disharmonious smile, impacting a patient's confidence. Many patients feel self‐conscious about their smiles, often inhibiting their full expression in various life situations.^7^

It is estimated that around 10% of individuals between the ages of 20 and 30 express concerns related to a gummy smile, with a higher prevalence observed among women.^8^, ^9^ Research has demonstrated that excessive gingival display affects patients' self‐esteem and influences their social and emotional relationships.^10^ Fortunately, excessive gingiva can be corrected through periodontal procedures, with or without the addition of restorative treatments.^8^ While many techniques have been described for crown lengthening in posterior regions for restorative and prosthetic purposes,^2^, ^11^, ^12^ there is limited discussion on the biopsychosocial impact of these procedures on patients, despite their growing popularity.

Therefore, this 2‐year follow‐up clinical study aims to explore the self‐assessed impact of ACL on the self‐esteem and quality of life of patients who undergo this procedure, as well as the gingival margin stability throughout the study follow‐up.

## MATERIALS AND METHODS

### Participant selection

The research protocol was submitted to the Institution's Research Ethics Committee (CAAE:22735519.9.0000.5418) and was conducted in accordance with the Helsinki Declaration. All participants provided informed consent. Patients seeking ACL surgery in the anterior maxilla who met the criteria for this procedure were selected based on the following:

#### Inclusion criteria

Patients had a diagnosis of periodontal health (gingival bleeding index and bleeding on probing <10%, clinical attachment level ≤3 mm) and a clinical indication for the procedure. This included an adequate amount of keratinized gingiva (at least 2 mm remaining after the surgical procedure planning) and a distance from the gingival margin to the CEJ ≥ 3 mm, associated with a gummy smile and/or short clinical crowns relative to the anatomical crowns.

#### Exclusion criteria

Exclusion applied to individuals with systemic conditions (e.g., diabetes, heart disease, and hepatitis) or those using medications (e.g., long‐term antibiotics, anti‐inflammatories, phenytoin, cyclosporine, and bisphosphonates) that could influence treatment response. Additional exclusion criteria included a diagnosis of gingivitis and/or periodontitis, smoking, pregnancy, or lactation.

To ensure accurate diagnosis, the following clinical parameters were assessed using a millimeter probe*. Plaque Index: presence or absence of biofilm^13^; Bleeding on Probing: presence or absence of bleeding on probing;^13^ Gingival Margin Position: Measured as the distance from the CEJ to the gingival margin; Probing Depth (PD): Measured as the distance from the gingival margin to the clinically detectable base of the periodontal pocket. Clinical examinations were done by a calibrated examiner (LSA, intra‐class correlation = 0.86 for PD).

### Self‐esteem and Oral Health Impact Profile – 14‐item evaluation

Before surgery, all patients received basic periodontal treatment, including supragingival scaling, prophylaxis, and oral hygiene instructions. Patients then completed the Oral Health Impact Profile – 14 items (OHIP‐14)/Brazil^14^ and the Rosenberg/UNIFESP‐EPM Self‐Esteem Scale^15^ questionnaires to assess their perceptions of self‐esteem and quality of life before and after surgery. The Rosenberg questionnaire addresses an individual's overall sense of self‐worth and self‐acceptance, evaluating both positive and negative self‐perception domains to provide a holistic view of their self‐esteem. It consists of 10 statements rated on a 4‐point Likert scale, with responses ranging from ‘Strongly Agree’ to ‘Strongly Disagree.’ Scores range from 0 to 30, where higher scores indicate greater self‐esteem, while lower scores suggest diminished self‐worth or a negative self‐view. The OHIP questionnaire was used to evaluate oral health impact. Both questionnaires’ summaries are presented in File S1. Patients were re‐evaluated in the clinic 30 days after surgery and completed the questionnaires online 2 years later.

### Surgical technique

Following clinical and radiographic examinations and case planning, surgical procedures were performed under local anesthesia with 2% lidocaine and 1:200,000 epinephrine by a single operator (RCVC). Figure 2A is the pre‐operatory clinical condition, showing the need for gingival margin correction. After anesthesia, the distance from the gingival margin to the CEJ was measured (Figure 2B), and these values were transferred to the gingival tissue to guide the procedure. Excess gingival tissue was removed using an internal bevel incision (Figure 2C,D), and, when necessary, an osteotomy was performed to re‐establish the supracrestal attachment tissue space (3 mm) (Figure 2E,F). Stabilization was achieved with internal vertical mattress sutures using 5‐0 nylon thread (Ethicon J&J) (Figure 2G), which were removed after 14 days. Analgesics (500 mg Dipyrone, every 6 hours) were prescribed for two days. Figure 2H is the patient's clinical condition after 2 years of the procedure. Figure 3 shows another clinical case included in this study, demonstrating, in sequence, how surgeries were performed and the final clinical outcome.

### Gingival phenotype and longitudinal clinical assessment

The gingival phenotype (regarding the evaluation of keratinized tissue and gingival thickness) was indirectly determined by the crown width/length ratio, as proposed by Fischer et al.^16^ and Zhong et al.^17^ The central incisors’ dimensions were obtained by measuring the intra‐oral photography at ImageJ software by a calibrated examiner (RCVC, ICC>85%). The crown length was measured at midline, and the width at the papillae line at baseline. Patients were categorized into thin or thick phenotypes: *thin*—when the crown width was less than 80% of its length; and *thick*—when the width/height ratio was greater than 80%. Also, the clinical crown length (CCL) at Baseline, 30‐day, and 2‐year follow‐up was compared to assess the gingival margin stability after surgery.

### Data analysis

OHIP‐14 and Rosenberg scale, as well as CCL data, were compared using Friedman's test. The Spearman correlation test was used to assess the association between Gingival Phenotype and ΔCCL at the 2‐year evaluation. A significance level of 5% was used for all statistical analyses.

## RESULTS

**FIGURE 1:**
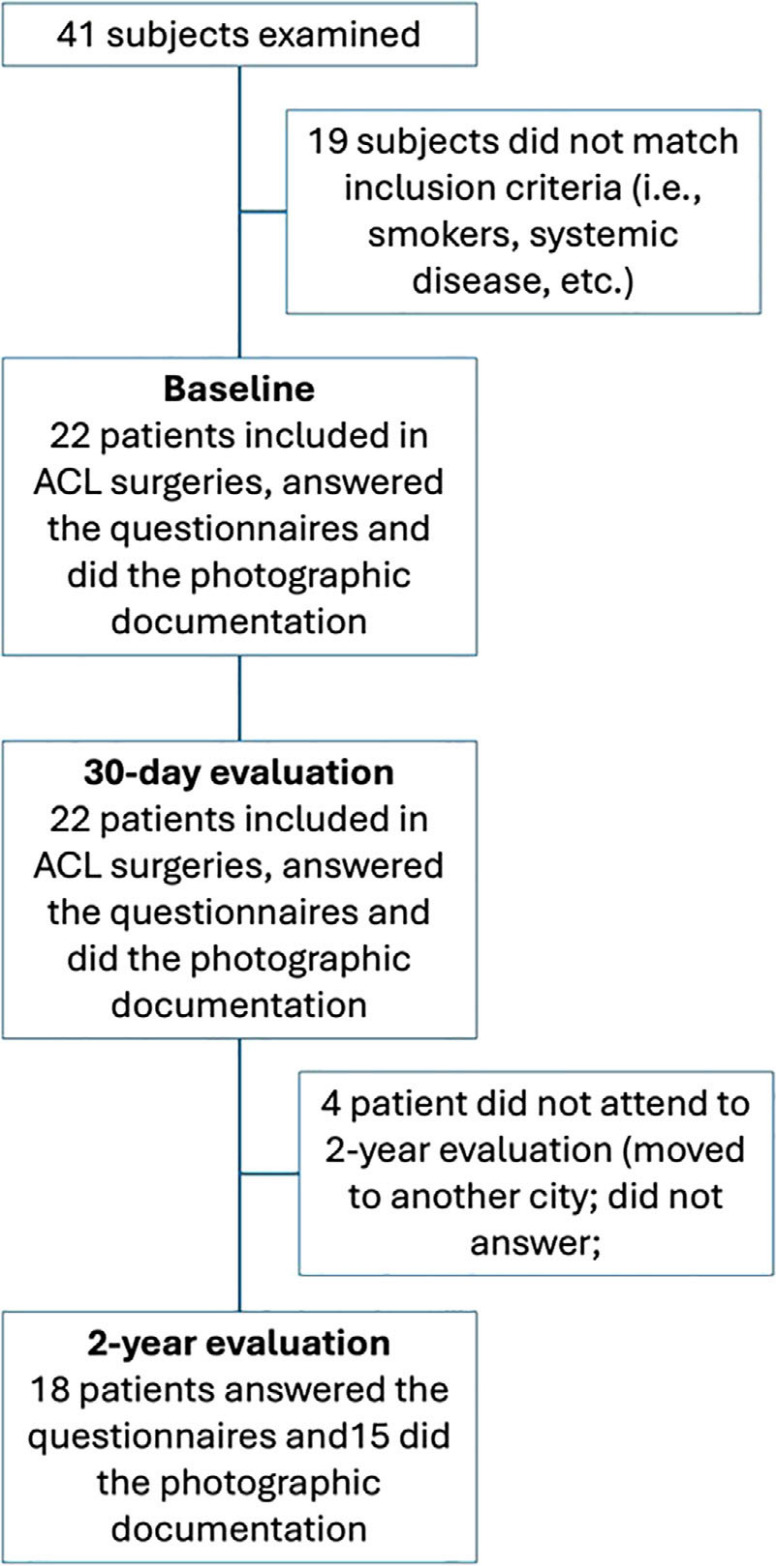
Flowchart of the study patient's inclusion.

The population (*n* = 22) included in this study was predominantly young (18–30 years old) and female sex (81.8%). No patient presented a probing depth of ≥5 mm, and BoP was lower than 20% to be assigned to ACL. All patients answered the questionnaires at baseline and 30 days, and 18 replied to them at the 2‐year assessment (Figure 1). Crown dimension analysis was performed only on those who completed the follow‐up and took intra‐oral photographs at the 2‐year follow‐up. A thick gingival phenotype was observed in 73% of cases that underwent ACL. CCL longitudinal analysis showed an increase after ACL (8.6 ± 0.8 mm at Baseline to 9.7 ± 1.8 mm after 30 days, *p* = 0.001) and stability in the gingival margin after 2 years (9.8 ± 2.1 mm), with no difference between 30‐day and 2‐year follow‐up (*p* = 0.34, Figure 4). The Gingival phenotype did not show a significant correlation with changes in CCL at 2 years (*r* = 0.2, *p* = 0.5).

**FIGURE 2:**
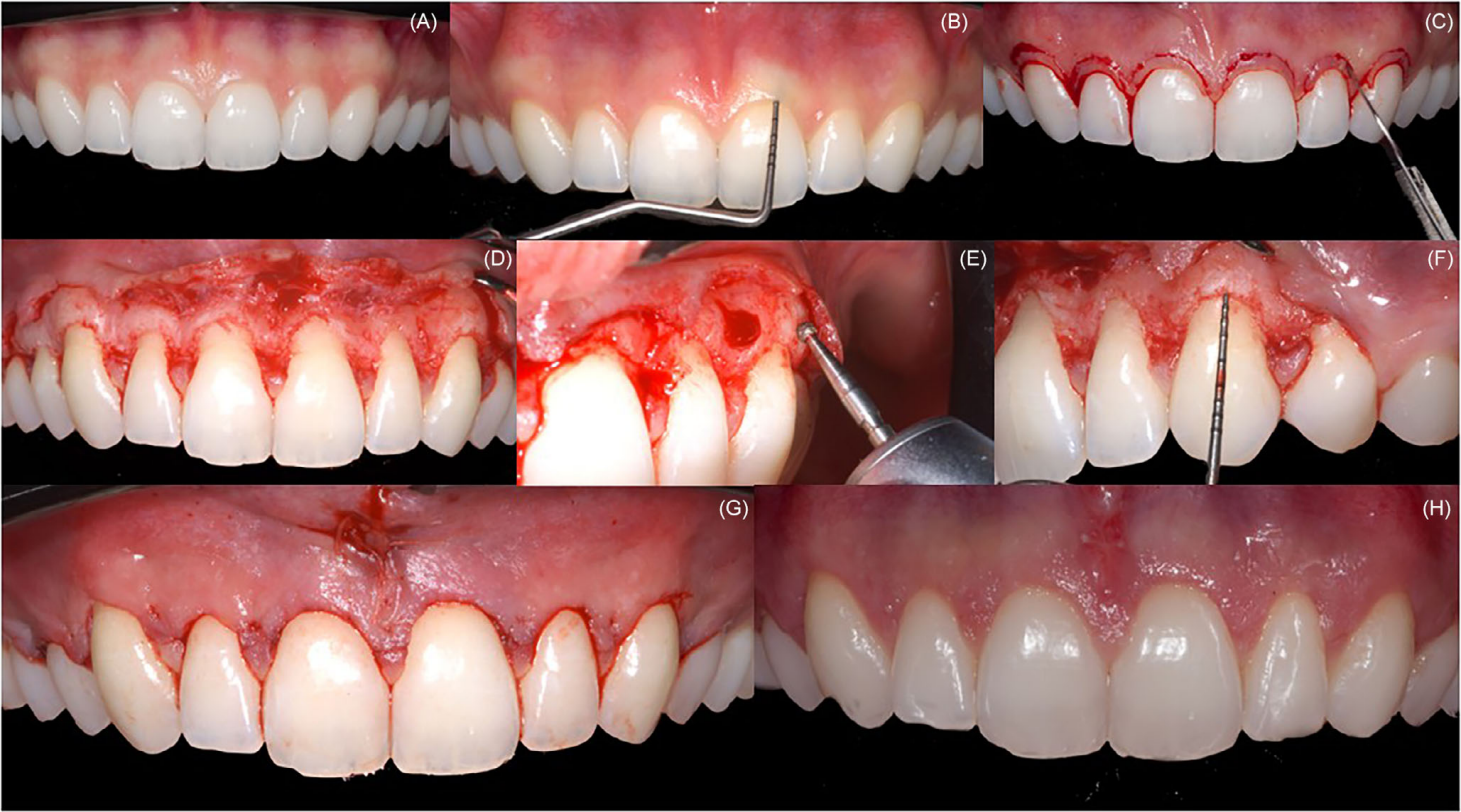
Pre‐operatory clinical condition (A), surgical steps (B–G), and clinical condition after 2 years of procedure (H).

**FIGURE 3:**
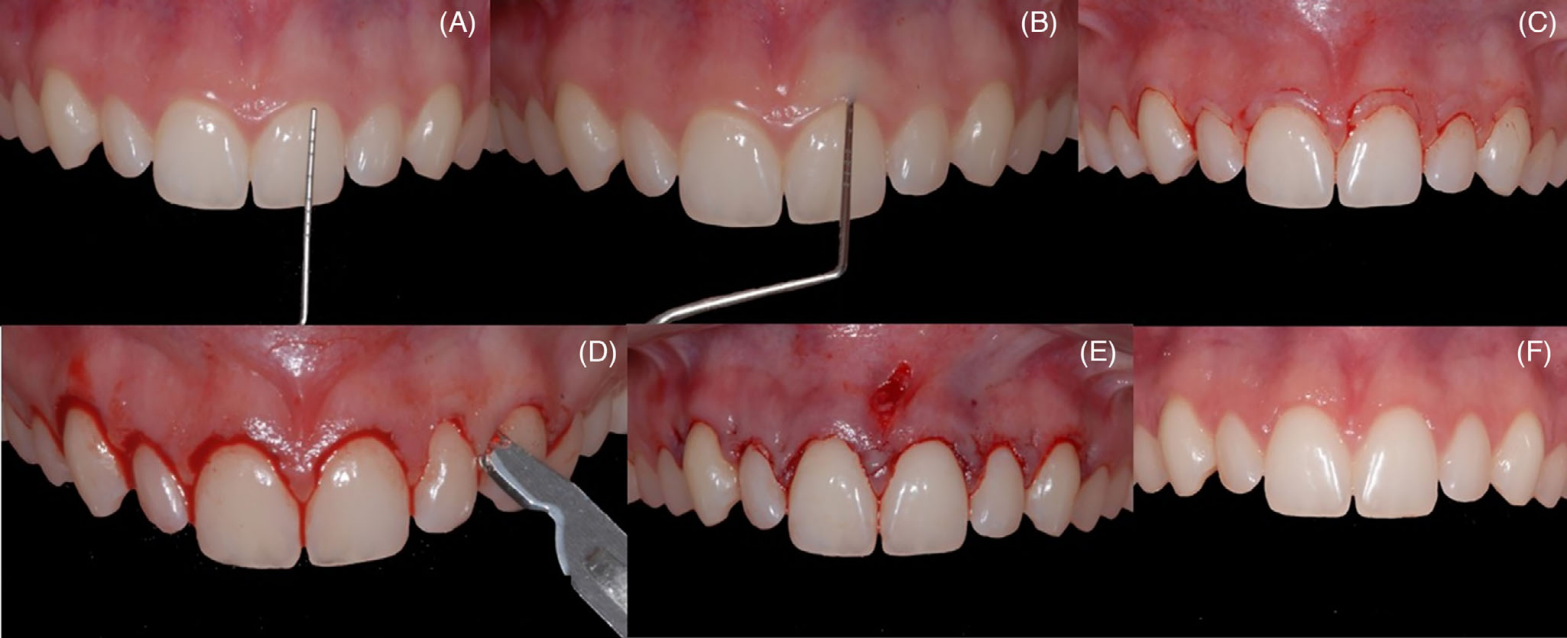
Pre‐operatory clinical condition (A), surgical steps (B–D), immediate postoperative condition (E), and final clinical outcome (F).

**FIGURE 4:**
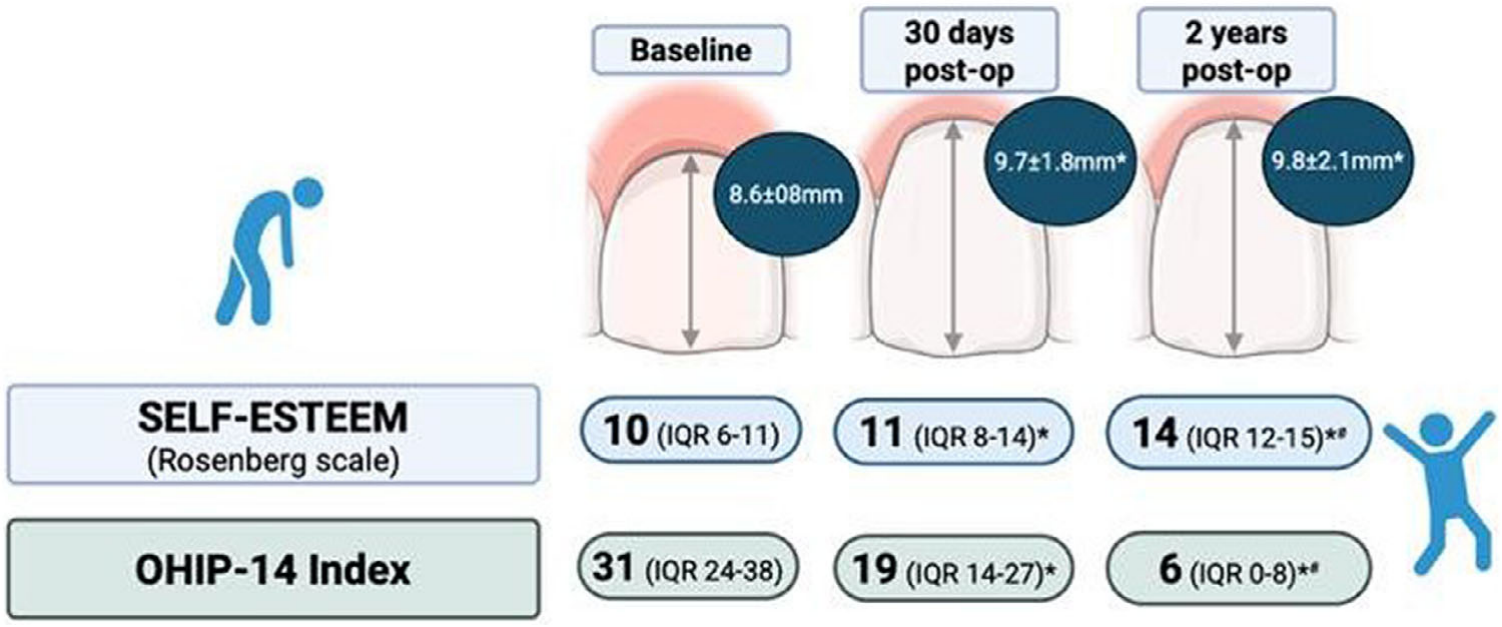
Median values (Interquartile Range) of Oral Health Impact Profile – 14 items (OHIP‐14) and Self‐esteem (Rosenberg Scale) questionnaire results at baseline, 30 days, and 2 years after ACL surgery and recurrence rate after 2 years postoperative. *Indicates statistically significant difference to baseline; ^#^indicates statistically significant difference to 30‐day evaluation (Friedman test, p < 0.05).

### Self‐esteem and OHIP analysis

**FIGURE 5:**
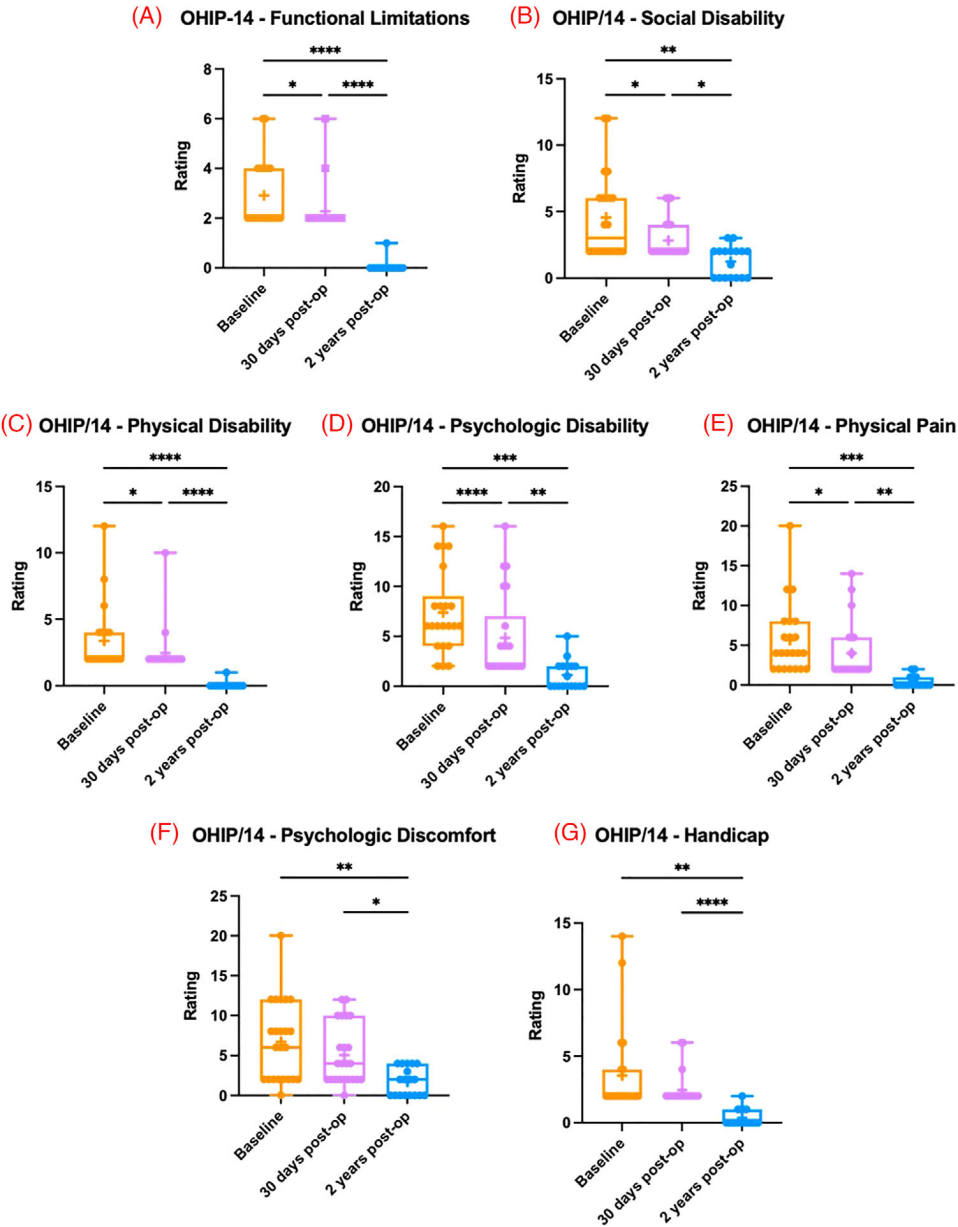
Average, median, and Interquartile Range of Oral Health Impact Profile – 14 items (OHIP‐14)/Brazil self‐esteem questionnaire results at baseline, 30 days post‐op and 2 years post‐op for the following categories: (A) functional limitations, (B) social disability, (C) physical disability, (D) psychologic disability, (E) physical pain, (F) psychologic discomfort, and (G) handicap. Statistical significance is indicated by *(p < 0.05), **(p < 0.01), ***(p < 0.001), and ****(p < 0.0001).

Along with the increased crown length dimensions, the assessment using the Rosenberg Self‐Esteem Questionnaire demonstrated a statistically significant enhancement in self‐esteem within the first 30 days post‐treatment (*p* = 0.024). Furthermore, a sustained and even greater improvement in self‐esteem was observed between the 30‐day and 2‐year follow‐ups (*p* = 0.003), highlighting the positive impact of the intervention on psychological well‐being (Figure 5).

An evaluation of the domains of the OHIP‐14 questionnaire at 30 days revealed a significant overall reduction in most assessed dimensions: functional limitations (*p* = 0.007), social disability (*p* = 0.001), physical disability (*p* = 0.002), physical pain (*p* = 0.001), psychological discomfort (*p* = 0.04), and handicap (*p* = 0.01). Psychological disability was the only parameter that remained unchanged (*p* = 0.82). After 2 years, all dimensions showed improvements, indicating an upgrade in oral health and quality of life (Figure 5).

## DISCUSSION

The present study evaluated the gingival margin dimensions following ACL surgery and its effects on self‐esteem and quality of life in patients with a gummy smile over a 2‐year period. Our findings indicate that significant improvements in self‐esteem and quality of life can be observed as early as 30 days post‐surgery. Furthermore, the clinical benefits persist for 2 years after the procedure, with an additional enhancement in self‐perception during this time. These results highlight the effectiveness of ACL in addressing aesthetic concerns associated with excessive gingival display while also contributing positively to patients' psychological well‐being and social interactions.

Mann et al. highlight that low self‐esteem is associated with risk behaviors and a greater predisposition to depression and anxiety.^18^ On the other hand, positive self‐esteem contributes to mental well‐being, happiness, personal success, and even better recovery from severe illnesses.^18^ In this sense, dental procedures that enhance smile aesthetics not only impact an individual's self‐perception but can also serve as a health‐promoting factor, preventing negative consequences associated with low self‐esteem. Beyond the clinical benefits, many patients who feel self‐conscious about their smiles may limit their social interactions or refrain from fully expressing themselves in various situations. Through patient‐reported outcomes, we demonstrated that ACL effectively addresses these concerns and significantly enhances quality of life. Notably, the surgery improved multiple dimensions of the OHIP‐14 index. Improvements were observed not only in physical dimensions (such as physical disability and pain) but also in psychological discomfort and social disability.

Some studies have highlighted the benefits of root coverage and periodontal regeneration treatments on quality of life.^19^, ^20^, ^21^ However, to the best of our knowledge, this is the first study confirming the impact of gingival smile correction on patient perceptions. Additionally, it is noteworthy that these improvements became even more pronounced after 2 years. This phenomenon may be attributed to an initial enhancement in self‐esteem and oral health‐related quality of life, which could lead to positive behavioral changes and psychological well‐being. Over time, these factors may contribute to sustained benefits in the individual's perception of their oral health and overall quality of life. Thus, periodontal plastic surgery goes beyond aesthetics, becoming a relevant part of comprehensive patient well‐being care. Furthermore, this study emphasizes the importance of individualized treatment plans that consider the specific anatomical and psychological needs of patients, as well as their impact on patient satisfaction.

In this study, 22 participants were included, most of whom were women (81.8%). This occurred because most of the patients seeking oral aesthetic procedures were female sex. Women commonly report a poorer quality of life related to oral health (greater impact, greater severity, and a higher prevalence of negative effects) than men. This is likely because women may be more aware of or more concerned about aesthetic aspects, pain, or discomfort, and this is reflected in their Questionnaire responses. This is also due to cultural factors that generate social expectations about appearance, smiles, and chewing function, which may weigh more heavily on women in many contexts.^22^, ^23^ Despite this, other demographic factors appear to have as significant an impact as sex on individuals' negative self‐perception of their oral health, such as family income (low), quality of life (low), social support (low, moderate), and smoking (smokers), as reported by Gabardo et al. (2015).^22^ This emphasizes that other factors, most of them socioeconomic, are as impactful as sex on OHIP‐14 outcomes.

Among the treatment options available for addressing a gummy smile, periodontal plastic surgery remains the most utilized procedure for correcting excessive gingival display, as greater gum exposure can negatively impact an individual's quality of life.^18^, ^24^ However, due to anatomical and phenotypic variations among patients, the surgical technique often requires modifications to achieve optimal aesthetic results while minimizing patient morbidity. The surgical protocol adopted in this study, which combines gingivectomy with ostectomy/osteoplasty, further supports the reliability of ACL as a viable treatment option, reinforcing previous findings in the literature.^1^, ^4^, ^24^, ^25^, ^26^, ^27^, ^28^, ^29^ It was demonstrated not only a predictable outcome over 2 years, but also the cost‐effectiveness of the technique, which ensures accessibility and makes it a sustainable option for improving patient outcomes.

Recently, Carrera et al.^30^ showed that the gingival thickness, measured at CBCT, did not show a significant correlation with gingival margin stability. Similarly, our findings revealed no association between gingival phenotype (determined by crown width/length ratio) and gingival margin changes after 2‐year follow‐up. It is important to highlight that most of the patients showed a thick gingival phenotype. While general evidence in the literature suggests that thicker tissues are more likely to be associated with post‐ACL tissue rebound,^1^, ^27^ no such association was seen in the recent^30^ and present study. This suggests that the effectiveness of ACL transcends phenotype classifications, reinforcing the notion that surgical outcomes may be consistent across different patient profiles. Moreover, although some studies suggest that thicker phenotypes may offer greater long‐term aesthetic stability,^4^, ^25^, ^26^ our data indicate that the impact of ACL is equally positive regardless of these variations, reinforcing the versatility and effectiveness of the approach for a wide range of patients. These results emphasize the importance of a comprehensive assessment, where the phenotype is just one factor to consider in developing individualized treatment plans, and not the sole determinant of the clinical and psychological success of the patient.

Another aspect to be considered is the gingival thickness measurement. Currently, the literature reports many ways to access the gingival phenotype, i.e., cone beam computed tomography (CBCT), Transparency on probing, etc. In 2020, Zhong et al. showed that the crown format can be associated with the gingival dimension. The study associated long, narrow crowns with a thin phenotype, while a short, wide crown was associated with the thick one.^17^ This is similar to what was reported by Stellini et al.,^31^ Fischer et al.,^16^ and Song et al.^32^ So, even though this is an easy method that can be applied routinely during surgical planning, clinicians can apply different ways to assess gingival thickness.

Meanwhile, it is important to consider that this is the first study assessing the psychological impact of ACL on self‐esteem and oral‐related quality of life. So, future studies will be able to determine the effect size and reach a sample size focused on specific measurements. However, the sample size of the present study (*n* = 22), despite being limited, was adequate to support the meaningful conclusions. Our sample size was determined based on prior clinical studies investigating similar clinical outcomes, ensuring that it is within the range commonly used in well‐conducted trials in this field.^30^, ^33^, ^34^, ^35^ Anyway, future exploratory clinical research in this field can include a larger and different population to confirm our results.

Finally, this study demonstrated that both the clinical benefits and patient‐reported outcomes of the procedure can be maintained over a 2‐year period, supporting its use as a valuable intervention in aesthetic periodontics. Further research is recommended to evaluate the long‐term effectiveness of this technique, considering both clinical outcomes and improvements in quality of life. Also, future analyses will be able to encompass more dimensions of psychological aspects, and the impact of specific variables on each domain, i.e., if the amount and severity of gingival display, before and after ACL, can influence the improvement of participants’ quality of life and self‐esteem.

## CONCLUSION

The study demonstrated that the ACL procedure effectively reduced gingival dimensions in the smile, with stable results after 2 years of follow‐up, and significantly improved the quality of life and self‐esteem of patients over the years. This highlights ACL as an excellent treatment option for addressing a gummy smile, offering both aesthetic and psychological benefits.

## AUTHOR CONTRIBUTIONS

**Leticia S. Arroteia**: Conceptualization (equal); investigation (equal); data curation (equal); writing—original draft preparation (lead). **Camila S. Stolf**: Conceptualization (lead); methodology (equal); formal analysis (equal); data curation (equal); writing—original draft preparation (equal). **Hélvis E. S. Paz**: Methodology (equal); formal analysis (lead); investigation (equal); data curation (equal); writing—original draft preparation (equal). **Thais F. M. Paschoal**: Software (lead); formal analysis (equal); investigation (equal). **Mabelle F. Monteiro**: Methodology (lead); validation; writing—review & editing (equal); funding acquisition (equal); project administration (equal). **Márcio Z. Casati**: Validation (equal); resources (equal); supervision (equal); funding acquisition (lead). **Mauro P. Santamaria**: Validation (equal); resources (equal); writing—review & editing (lead). **Renato C. V. Casarin**: Conceptualization (lead); methodology (equal); formal analysis (equal); visualization (equal); writing—review & editing (equal); project administration (lead).

## CONFLICT OF INTEREST STATEMENT

The authors declare no conflicts of interest.

## Supporting information



Supporting Information

## Footnotes

* North Carolina Probe PCP 15, Hu‐Friedy, Chicago, IL, USA
