# Supplementary material for: Effect of aesthetic crown lengthening on life quality and self‐esteem: 2‐year study
Source: Clin Adv Periodontics. 2026 May 22;16(3):703–11. doi: 10.1002/cap.70030 (PMC13603051; doi:10.1002/cap.70030)
Supplement: Supplementary file 1 [file CAP-16-703-s001.docx]

**Supplementary File 1: Questionnaires’ summaries.**

A) **Brazilian version of the short-form of the Oral Health Impact Profile (OHIP-14/Brazil):**

Nos últimos seis meses, por causa de problemas com seus dentes, sua boca ou dentadura:

1 – Você teve problemas para falar alguma palavra?

2 – Você sentiu que o sabor dos alimentos tem piorado?

3 – Você sentiu dores em sua boca ou nos seus dentes?

4 – Você se sentiu incomodada ao comer algum alimento?

5 – Você ficou preocupada?

6 – Você se sentiu estressada?

7 – Sua alimentação ficou prejudicada?

8 – Você teve que parar suas refeições?

9 – Você encontrou dificuldade para relaxar?

10 – Você se sentiu envergonhada?

11 – Você ficou irritada com outras pessoas?

12 – Você teve dificuldade para realizar suas atividades diárias?

13 – Você sentiu que a vida, em geral, ficou pior?

14 – Você ficou totalmente incapaz de fazer suas atividades diárias?

Opções de respostas: Nunca (0), Raramente (1), Às vezes (2), Repetidamente (3) e Sempre (4).

B) **Rosenberg/UNIFESP-EPM Self-Esteem Scale:**

1 – De uma forma geral (apesar de tudo), estou satisfeito comigo mesmo (a).

2 - Às vezes, eu acho que não sirvo para nada (desqualificado ou inferior em relação aos outros).

3- Eu sinto que eu tenho um tanto (um número) de boas qualidades.

4 - Eu sou capaz de fazer coisas tão bem quanto a maioria das outras pessoas (desde que me ensinadas).

5 - Não sinto satisfação nas coisas que realizei. Eu sinto que não tenho muito do que me orgulhar.

6 - Às vezes, eu realmente me sinto inútil (incapaz de fazer as coisas).

7 - Eu sinto que sou uma pessoa de valor, pelo menos num plano igual (num mesmo nível) às outras pessoas.

8 - Eu gostaria de ter mais respeito por mim mesmo (a). (Dar-me mais valor).

9 - Quase sempre, estou inclinado (a) a achar que sou um (a) fracassado (a).

10 - Eu tenho uma atitude positiva (pensamentos, atos e sentimentos positivos) com relação a mim mesmo (a).

Opções de respostas para as perguntas 1, 3, 5, 7 e 9: Concordo plenamente (0), Concordo (1), Discordo (2), Discordo plenamente (3).

Opções de respostas para as perguntas 2, 4, 6, 8 e 10: Discordo plenamente (0), Discordo (1), Concordo (2), Concordo plenamente (3).
